# 

**Published:** 1912-02-24

**Authors:** 


### The Ætiology of Leprosy.

A short time ago an account was given of the views formed by a research worker among the lepers of Robben Island, where the lepers of Cape Colony are segregated. This author attributed to the bed-bug the transference of the organism of leprosy from affected to healthy persons. In the *British Journal of Dermatology*, Dr. Turner, of Gibraltar, describes a case of leprosy whose origin is most mysterious; indeed it is difficult to see how any theory of contagion from a pre-existing form can account for the facts. A native of Gibraltar, who had been imbecile from birth, was admitted to the Gibraltar Lunatic Asylum by request of his family in 1883, at the age of twenty-eight. His parents died at advanced ages, and all his relatives can be acquitted of leprosy, though several of them show stigmata of mental degeneration. For nineteen years this patient remained healthy in the asylum, but then in 1902 he developed thickenings of the hands and feet. It was not until a year later that a diagnosis of leprosy was thought of; but once suggested, it was soon abundantly proved bacteriologically. The patient died of his disease in 1906; how did he acquire it? The author states that of his own knowledge no case of leprosy has been known in Gibraltar for more than thirty years; for nineteen years previous to the outbreak of symptoms his only communication with the outside world was a visit from his sisters of half-an-hour once a fortnight. The asylum occupies an isolated site, fenced by high walls out of which the patients never pass. Now comes an interesting point. On investigating the dietary of the asylum, Dr. Turner found that from 1900 to 1903 fish was issued twice a week; one ration was of fresh, and one of salt fish. The coincidence is interesting in view of Sir J. Hutchinson's well-known hypothesis, though it is not likely that it will reverse the adverse verdict with which those views have been received by the profession.

### Buildings Contemplated and in Progress.

Acton.—The Health Committee has instructed the Surveyor to prepare working drawings, etc., for an administrative block at the Isolation Hospital at an estimated cost of £3,400.

Bangor.—Tenders are invited by the Guardians for the erection of a new infirmary at Bangor, North Wales. The design has been prepared by the Guardians' architect, Mr. Frank Bellis.

Barking.—The Urban Council have received six tenders for the erection of an administrative block at the Upney Infectious Hospital. The lowest is £1,630, the highest £2,197.

Barrow.—A new laundry is projected at the North Lonsdale Hospital, Barrow. Mr. H. T. Fowler, A.R.I.B.A., is the architect, and tenders are now invited.

Bucknall.—The Joint Hospital Board met at Hanley to discuss the question of increased accommodation for nurses. It was decided to provide twenty-two additional bedrooms and the usual day-rooms at an estimated cost of £2,400.

Cardiff.—Tenders are invited for supplying a new boiler for the Cardiff Workhouse, of Galloway, Lancashire or Yorkshire type, with standard fittings and of dimensions 8.0 by 30.0, to be insured at a working pressure of 100 lb. to the square inch.

Chandlersford.—Tenders are invited for an Infectious Hospital at Fryern Hill. Applications should be made to the Council's Surveyor at Eastleigh.

Chester.—Alterations and extensions to the Chester Infirmary advised by Mr. Paul Waterhouse, F.R.I.B.A., are to be carried out under the direction of Mr. Wm. T. Lockwood, F.R.I.B.A., Architect. Tenders are now invited for the works.

Glasgow.—Mr. J. J. Burnet, A.R.S.A., is architect for the Royal Hospital for Children, in course of erection at Yorkhill. It is hoped that the buildings will be completed in 1914. The cost is estimated at £140,000.

London.—The Guardians of St. Giles, Camberwell, have received tenders for additional accommodation for nurses at their workhouse according to the plans and specifications prepared by Mr. A. E. Mullins, their architect. Twenty-six firms submitted estimates, varying from £3,135 to £1,998.

Macclesfield.—The Governors of the Macclesfield Infirmary contemplate structural alterations and improvements to the buildings under their charge, but full details are not yet issued.

Neath.—Mr. J. C. Rees is architect for extensive works at the Neath Infirmary, including the erection of an administrative block, pavilions for sick and infirm children's home, porter's lodge and mental wards, laundry engine-house, nurses' home, and roads, sewers, etc. Estimates are to be delivered by February 27.

Nordrach-on-Dee.—Extensions are being carried out to the Sanatorium, at a cost of £3,000, by Mr. W. E. Gauld, of Aberdeen.

Redruth.—It is proposed to expend £2,000 on alterations and additions to the Infirmary. Particulars from the Clerk to the Guardians, Redruth.

South Shields.—The Guardians are negotiating with the Harton Coal Company, Ltd., for the acquisition of West Hall, to be used as a consumption hospital.

Stoke-on-Trent.—For additions to the children's ward at the North Staffs Infirmary, six tenders have been received, varying from £2,810 to £2,600. Messrs. Scrivener and Son, of Hanley, are the architects.

Uxbridge.—The Guardians have accepted a tender by Messrs. Hayward Bros. and Eckstein, Ltd., of £6,982 for heating, etc., at the workhouse.

Weybridge.—An enlargement of the Weybridge Cottage Hospital is proposed. Particulars may be had from the architect, care of the Trustees.

Winchester.—Drawings of proposed nurses' quarters and maternity wards, to be erected at the Union Workhouse, may be seen at the office of the architect, Mr. B. D. Cancellor, Winchester.

Winsley, Bradford-on-Avon.—Extensions are proposed at the Sanatorium at a cost of £9,000. Particulars may be had from Mr. W. S. Skinner, Baldwin Street, Bristol.

### Appointments.

MR. H. H. FYFFE has been appointed Junior Resident Medical Officer at the Royal Infirmary, Sheffield.

DR. FREDERIC FRANCIS GERMAN has been appointed Medical Officer of Health for the Seaforth urban district, Liverpool, subject to the approval of the Local Government Board.

A VACANCY.

THE post of House Surgeon to the Dewsbury and District Hospital is now vacant, owing to the resignation of Dr. Storey, after two and a half years' work. Dr. Fittar and the hospital's president both spoke in the warmest terms of Dr. Storey's work.

---

MR. WILLIAM PRICE, a working man, residing in Cardiff, has given a donation of £100 towards the Memorial Fund of the Cardiff Hospital. Special attention was called to this donation at the recent monthly meeting.

### Florence Nightingale Memorial.

Received by Mr. G. Q. Roberts: Australasian Trained Nurses' Association, £74; St. George's Hospital Collection, £25; J. Gerstley, Esq., £5 5s.; C. D. Montefiore, Esq., £5; Miss C. Henderson's Collection, £2 10s.; Mrs. J. Halpin, £2 2s.; Mrs. Smith Lewis, Litt.D., £2 2s.; Miss Augusta Spottiswoode, £2 2s.; H. H. Wills, Esq., £2 2s.; A. C. Bradley, Esq., £2; Lieut.-Colonel A. T. Moore, R.E., £2; Mrs. Gibson, Litt.D., £1 1s.; L. M. Wynch, Esq., £1 1s.; Sidney E. R. Lane, Esq., £1 1s.; Colonel J. G. Kelly, £1 1s.; E. Laurie Robinson, Esq., £1 1s.; General the Hon. Sir Savage Lloyd Mostyn, £1; Mrs. Bramall, £1; Mrs. Smith, £1; Colonel Harold Wylly, 10s. 6d.; Major A. Lloyd, 10s. 6d.; Dr. George Rice, 10s. 6d.; Miss E. F. Gibbs (annuities), 5s.; Mrs. Thorneycroft, 5s.; Mrs. T. G. Wood, 5s.; Miss Barretti, 3s.; From fourteen Indian Mutiny and Crimean Veterans, 2s. 6d.

